# Partial Rhombencephalosynapsis Presenting in an Adult with Cerebello-Trigeminal-Dermal Dysplasia

**DOI:** 10.1016/j.ebr.2024.100688

**Published:** 2024-06-22

**Authors:** Frances Tiffany Cava Morden, Bao Xin Liang, Linda Nguyen, Enrique Carrazana, Arash Ghaffari-Rafi, Kore Kai Liow

**Affiliations:** a University of Hawaiʻi at Mānoa, John A. Burns School of Medicine, Honolulu, HI, USA; b Hawaii Pacific Neuroscience, Honolulu, HI, USA; c Neurelis, Inc., San Diego, CA, USA; d University of California, Davis, School of Medicine, Department of Neurological Surgery, Sacramento, CA, USA

**Keywords:** Rhombencephalosynapsis, Cerebello-trigeminal-dermal dysplasia, Gomez-Lopez-Hernandez syndrome, Cerebellar dysfunction, First-time onset seizures, Adult

## Abstract

Gomez-Lopez-Hernandez syndrome (GLHS), also known as cerebello-trigeminal-dermal dysplasia, is a neurocutaneous disorder typically presenting in childhood. GLHS is characterized by rhombencephalosynapsis (RES) and partial alopecia, with or without trigeminal anesthesia. We describe a rare case of GLHS in a paucisymptomatic adult who presented with new-onset seizure-like activity. Magnetic resonance imaging revealed partial midline fusion of the cerebellar hemispheres, incomplete development of vermis, and slight medialization of the dentate nuclei: all consistent with the diagnosis of RES. Radiographic evidence combined with partial alopecia, truncal ataxia, and muscular hypotonia are suggestive GLHS diagnosis. Our report not only highlights the importance of maintaining GLHS on the differential for new-onset seizure-like activity, but also demonstrates how patients with GLHS may be minimally symptomatic and diagnosed in adulthood.

**Lay Summary:**

The Gomez-Lopez-Hernandez syndrome (GLHS), or cerebellotrigeminal-dermal dysplasia, is a rare condition that affects both the nervous system and the skin. It involves abnormal development of the brain, partial alopecia [thinning of hair], and loss of sensation in the face. One specific brain malformation, called rhombencephalosynapsis (RES), results from abnormal formation of the cerebellum and is seen in GHLS.Both RES and GLHS present early in childhood, and cases presenting later in life are exceptionally rare. Here we describe a young adult with RES and GLHS whose normal development and mild clumsiness eluded recognition by doctors until early adulthood when she presented with a single seizure.

## Highlights

• Gomez-Lopez-Hernandez syndrome (GLHS) patients may be paucisymptomatic.
• GLHS may be diagnosed in adulthood.
• New-onset adulthood seizures may be the first presenting symptom of GLHS recognized.
• Clinically GLHS is defined by: partial alopecia, truncal ataxia, muscular hypotonia.
• GLHS exhibits: cerebellar fusion, vermian immaturity, dentate medialization.

## Introduction

In 1979, Dr. Manuel Gomez was the first to describe Gomez-Lopez-Hernandez syndrome (GLHS), also known as cerebello-trigeminal-dermal dysplasia, in a female child with brachycephaly, hypertelorism, convergent strabismus, alopecia, truncal ataxia, and trigeminal anesthesia [1]. GLHS is a rare neurocutaneous condition traditionally characterized by the triad of rhombencephalosynapsis (RES), partial alopecia, and trigeminal anesthesia; however, previous studies have found that trigeminal anesthesia is not always present [2]. Since its discovery in 1979, there have been approximately 60 reported cases of the syndrome, with nearly all patients presenting prior to adulthood [3]. Despite this, its etiology is still unknown. Most of the literature has centered on theories regarding the etiology of RES, which is characterized by midline fusion of the cerebellar hemispheres and partial or total hypoplasia of the vermis [4]. Because both GLHS and RES primarily present in childhood, etiological theories have focused on genetic mutations or other disruptions in the pathways controlling the normal embryological development of the cerebellum and vermis.

Here, we describe a rare manifestation of GLHS in an adult with RES, partial alopecia, and other characteristics consistent with the condition. To our knowledge, this is the first case of GLHS in the Pacific Island region and one of only several cases presenting in an adult.

## Case description

We present a 22-year-old female with a history of anemia and attention deficit hyperactivity disorder (ADHD) who arrived to our neurology clinic for a new-onset seizure, which occurred three months prior. In July 2022, the young woman was instructed to go to the emergency department for low hemoglobin attributed to heavy menstrual bleeding. Other laboratory testing was insignificant. After receiving a blood transfusion, she was noted to have seizure-like activity—right leg and right arm tremors without loss of consciousness for 30 s. She described an aura of feeling weak. The woman and family denied any post-ictal state, confusion, or tongue-biting. She was born after a normal pregnancy from non-consanguineous parents and had a developmentally uneventful childhood. There was no family history of neurological disorders. On clinical exam, no dysmorphic features were noted.

**Fig. 1:**
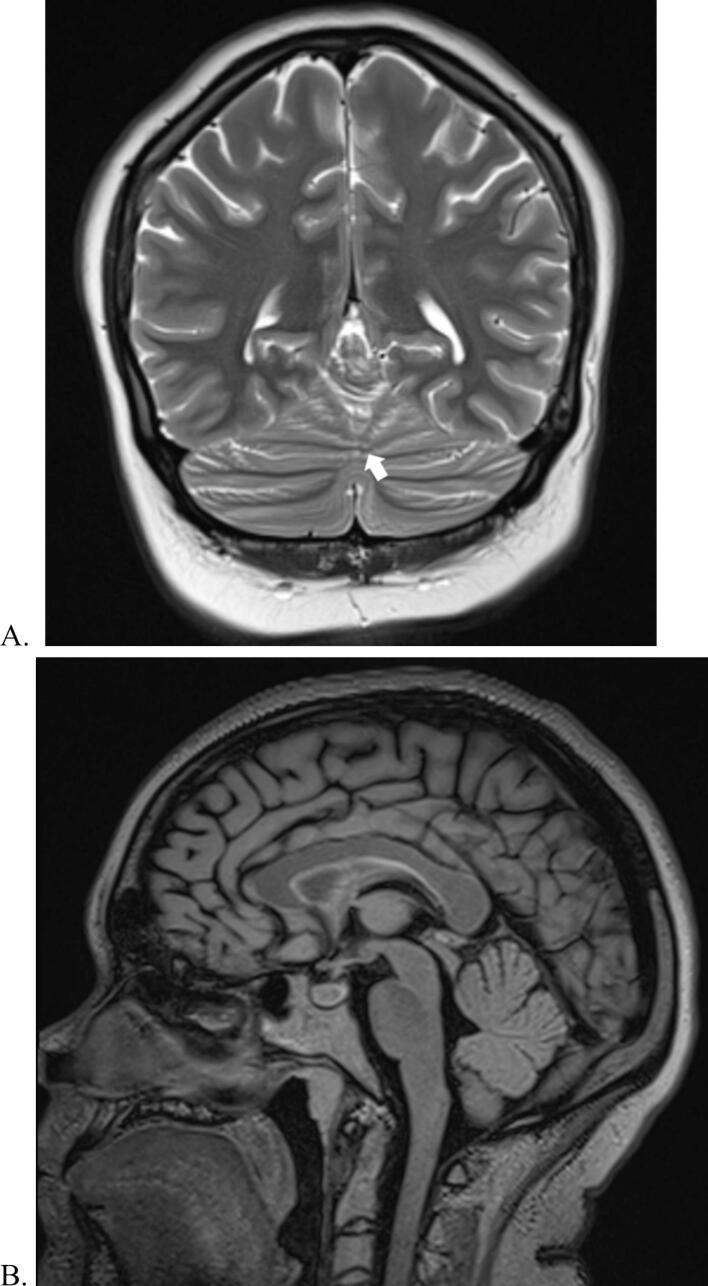

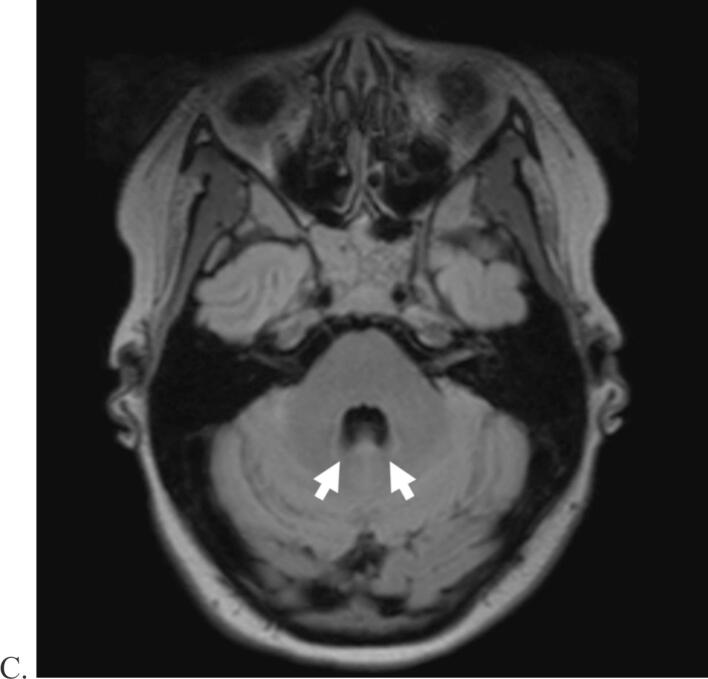
Magnetic resonance imaging of our patient. A) Coronal T2-weighted sequence showing partial midline fusion of the cerebellar hemispheres with continuity of the folia (white arrow). B) Sagittal T2-weighted flair sequence showing subtle incomplete development of vermis. C). Axial T2-weighted flair sequence showing slight medialization of the dentate nuclei (white arrows).

The neurological examination revealed mild truncal ataxia (during gait evaluation) and muscular hypotonia, with normal cognition, head shape, cranial nerve function, and muscle strength. Physical exam was notable for partial parieto-temporal alopecia. Magnetic resonance imaging (MRI) demonstrated a partial midline fusion of the cerebellar hemispheres with mild medial displacement of the dentate nuclei and incomplete development of the cerebellar vermis (Fig. 1). Otherwise, the woman had normal cerebral cortices, sulci, ventricles, and corpus callosum development. An electroencephalogram (EEG) revealed poorly developed posterior alpha rhythms with an increase of theta frequencies over temporal areas bilaterally. Overall, the EEG demonstrated a diffusely disorganized background without focal abnormalities or epileptiform features.

## Discussion

Our diagnosis of partial RES was based on the MRI findings of partial cerebellar fusion, medialization of the dentate nuclei, and hypoplasia of the vermis, along with her clinical features of mild truncal ataxia, muscular hypotonia, and ADHD, are all consistent with previous literature [1], [2], [3], [4], [5]. RES is often associated with congenital conditions including GLHS, VACTERL, and Chiari malformations or may be isolated [4], [5]. Of note, other central nervous system abnormalities associated with RES were not found in our case, such as: hypoplasia of the cerebellar vermis, fusion of the thalami, temporal lobe hypoplasia, incomplete hippocampal inversion, hypoplasia of the optic, aqueductal stenosis, or hydrocephalus. Partial RES in addition to the partial parieto-temporal alopecia is suggestive of a low-spectrum GLHS by previous diagnostic criteria [7]. Traditionally, GLHS was diagnosed with a triad of RES, alopecia, and trigeminal anesthesia; however, studies have found that trigeminal anesthesia is not always present in this syndrome, as was in our case [2].

To the best of our knowledge, this is one of only several cases of GLHS presenting as a developmentally normal adult [8]. There have been several studies of RES presenting in adults without neurocutaneous abnormalities. Our patient has been relatively unaffected by her condition, likely accounting for her late presentation and diagnosis. Nevertheless, she did exhibit other clinical features known to be associated with RES including attention deficit hyperactivity symptoms, mild truncal ataxia, and mild hypotonia [4], [5], [9]. These clinical characteristics also correlate to her MRI findings of cerebellar fusion and hypoplastic vermis. Previous studies have found cerebellar dysfunction to be associated with cognitive dysfunction including attention deficit hyperactivity spectrum disorders [9]. Similarly, vermian hypoplasia has been previously associated with truncal ataxia and hypotonia [9]. Our patient presented with new-onset seizure-like activity with no focal abnormalities on initial EEG, but was not formally diagnosed with a seizure disorder due to refusal for further testing. Interestingly, there has been a previous report of RES presenting with focal impaired awareness seizure in a teenage patient [9].

The etiology of GLHS or RES is still unclear. Several genes have been implicated in the development of RES. Experimental studies have elucidated that the expression of homeobox genes Otx2 and Gx2 respectively define the caudal and rostral limits of the “isthmic organizer” of the cerebellum [10]. This isthmus is located between the mesencephalon that develops into the vermis and the metencephalon that becomes the cerebellar hemispheres. In addition, Sarnat [6] suggested that RES is caused by the underexpression of a gene involved in dorsal–ventral organization. A recent molecular study of 13 Brazilian patients with GLHS found no common gene that may be causing the condition [7]. Another hypothesis proposes RES be caused by a defect in the migration or a loss of the anterior cerebellar anlage cells pre-destined to form the vermis [8]. Given our patient’s mild presentation of the condition, it is supportive of variable expression of a genetic basis for the condition, or a late insult in the process of cerebellar and vermian formation.

## Conclusion

This is a rare case of GLHS presenting as a paucisymptomatic adult. Previous cases of GLHS had primarily been diagnosed in the pediatric population. This patient’s delayed presentation of RES-associated neurological manifestations of non-epileptic seizure, truncal ataxia, muscular hypotonia, and ADHD, along with partial alopecia, emphasizes the wide range of GLHS presentations. Physicians should keep in mind the broad spectrum of severity of GLHS signs and symptoms, with milder forms potentially delaying diagnosis until adulthood.

## Ethical Declarations

### Author contributions

All authors contributed equally to the development of the manuscript.

## Funding

None.

### Ethics approval

Institutional review board approval was waived by the University of Hawai‘i, Office of Research Compliance.

### Informed Consent Statement

Not applicable.

### Code Availability

Not applicable.

### Consent to Participate

Not applicable.

### Consent for Publication

All authors have read and agreed to the published version of the manuscript.

### CRediT authorship contribution statement

**Frances Tiffany Cava Morden:** Writing – review & editing, Writing – original draft, Project administration, Methodology, Investigation, Conceptualization. **Bao Xin Liang:** Writing – review & editing, Writing – original draft, Data curation, Conceptualization. **Linda Nguyen:** Writing – review & editing, Writing – original draft, Data curation, Conceptualization. **Enrique Carrazana:** Writing – review & editing, Writing – original draft, Investigation, Funding acquisition, Formal analysis, Data curation, Conceptualization. **Arash Ghaffari-Rafi:** Writing – review & editing, Writing – original draft, Formal analysis, Data curation, Conceptualization. **Kore Kai Liow:** Writing – review & editing, Writing – original draft, Visualization, Validation, Supervision, Resources, Project administration, Methodology, Investigation, Funding acquisition, Formal analysis, Data curation, Conceptualization.

## Declaration of competing interest

The authors declare that they have no known competing financial interests or personal relationships that could have appeared to influence the work reported in this paper.
